# ¿Debemos adaptarnos los laboratorios clínicos a la realidad del paciente con enfermedad renal crónica en la cuantificación de la hormona paratiroidea?

**DOI:** 10.1515/almed-2020-0127

**Published:** 2021-04-13

**Authors:** María Luisa González-Casaus, Pilar Fernández-Calle, Antonio Buño Soto

**Affiliations:** Análisis Clínicos, Hospital central de la Defensa Gomez Ulla, Madrid, España; Análisis Clínicos, Hospital Universitario La Paz, Madrid, España

**Keywords:** enfermedad renal crónica (ERC), hormona paratiroidea (PTH) biointacta, PTH intacta, variabilidad analítica, variación biológica

## Abstract

**Introducción:**

La aportación del Laboratorio Clínico en el ámbito diagnóstico es cada día mas importante porque gran parte de las decisiones clínicas que se adoptan se basan en nuestros resultados.

**Contenido:**

La cuantificación en sangre de hormona paratiroidea (PTH) presenta una importante variabilidad analítica debido a la heterogeneidad de sus formas circulantes y a la configuración antigénica de los diferentes métodos disponibles. Esta circunstancia puede tener impacto en aquellas situaciones patológicas que cursan con valores circulantes de PTH excesivamente elevados, como sucede en la enfermedad renal crónica (ERC).

**Resumen:**

A pesar de la identificación de otras moléculas involucradas en las alteraciones óseas y minerales asociadas a la ERC, como el klotho o el factor fibroblástico 23, los nefrólogos siguen basando sus decisiones terapéuticas en la PTH; el problema es que, el desconocimiento de estos aspectos analíticos en su cuantificación, puede inducir a errores en la interpretación clínica de sus resultados.

**Perspectiva:**

Esta revisión aborda estas consideraciones desde el Laboratorio Clínico y plantea posibles estrategias futuras, que afectan tanto a la elección del método como a la expresión de los resultados de PTH, con la finalidad de acercarnos más a la realidad del paciente renal, en colaboración con el nefrólogo.

## Introducción

La aportación del Laboratorio Clínico en el diagnóstico y seguimiento de múltiples patologías es cada día mas importante ya que gran parte de las decisiones clínicas que se adoptan se basan en nuestros resultados. Por ello, el conocimiento de las características de la magnitud que estamos analizando es crucial, no solo para interpretar adecuadamente los resultados, sino también para evaluar qué metodología es la mas idónea y proporciona mayor información. La cuantificación de la hormona paratiroidea (PTH), herramienta básica para evaluar las alteraciones de la homeostasis mineral, representa un claro ejemplo de ello.

El principal problema que plantea la cuantificación en suero o plasma de PTH es la gran variabilidad en sus resultados cuyo desconocimiento puede dar lugar a errores de interpretación y decisiones inadecuadas. Gran parte de esta variabilidad, como en muchas otras magnitudes analíticas, depende de condicionamientos preanalíticos inherentes a su biología y al manejo de la muestra. Sin embargo, en el caso particular de la PTH, existen importantes aspectos analíticos que son responsables de una gran variabilidad inter-método y que pueden constituir una potencial fuente de error en el diagnóstico, especialmente en aquellas patologías que cursan con niveles circulantes de PTH excesivamente elevados, como sucede en la enfermedad renal crónica (ERC). Estas circunstancias no solo dificultan la comparabilidad de resultados entre diferentes laboratorios, sino que además condicionan que en la interpretación de los resultados de PTH se deban valorar “tendencias”, mas que valores absolutos.

## Factores que contribuyen a la variabilidad pre-analítica de la PTH

La PTH presenta una variación biológica importante (raza, edad, índice de masa corporal [IMC]), está sujeta a ritmo circadiano y es muy sensible a pequeñas modificaciones en la calcemia, ya que el calcio ionizado extracelular es el principal regulador de la secreción y síntesis de la PTH. Las situaciones de hipocalcemia estimulan, en segundos, la secreción de PTH a través de un receptor-sensor de calcio [1] en la membrana de las células paratiroideas, mientras que la hipercalcemia inhibe rápidamente su liberación y favorece su degradación. De este modo, es impensable evaluar correctamente un resultado de PTH sin conocer el valor de la calcemia.

Junto con el calcio ionizado, participan otros factores en la función paratiroidea: el fósforo [2], el calcitriol (1,25-dihidroxivitamina D) [3] y el factor de crecimiento fibroblástico 23 (FGF23) [4]. La PTH regula la homeóstasis del calcio incrementando la resorción ósea, la reabsorción tubular de calcio e incrementando la síntesis renal de vitamina D. Por ello, es importante tener en cuenta que las situaciones de hipovitaminosis D, muy prevalentes, influyen en los resultados de PTH al ocasionar un hiperparatiroidismo secundario; de hecho, el valor umbral del calcidiol circulante (25-hidroxivitamina D, marcador de reserva) a partir del cual se incrementa la secreción de PTH para mantener los valores de calcitriol (vitamina D activa) en rango fisiológico, es uno de los criterios bioquímicos utilizados para definir la deficiencia de vitamina D. Posiblemente, los valores inferiores de calcidiol sérico que se registran en la raza negra justifica los resultados mas elevados de PTH que presentan en comparación con la población blanca.

Por otra parte, la utilización de suplementos de biotina puede interferir en los resultados dependiendo del método de inmunoensayo utilizado [5]. Pero sobre todo es importante tener en cuenta que, tanto la matriz biológica utilizada para su medición como la conservación de la muestra, influyen en los resultados de la PTH; es mas estable en plasma que en suero y a 4 °C que a temperatura ambiente. El grupo de trabajo de PTH de la *International Federation of Clinical Chemistry* (IFCC) [6] recomienda que en la medición de PTH en plasma EDTA, se separe el plasma antes de las 24 h tras la venopunción y se analice dentro de las 72 h si se conserva a 4 °C. La utilización de suero puede ser de elección si se analiza entre las 3–4 h tras la extracción o se conserva a −20 °C ya que presenta la ventaja de poder cuantificar el calcio simultáneamente. Hay algún estudio [7] que demuestra que la utilización de tubos que contienen trombina para una separación rápida del suero (tubos RST, del inglés Rapid serum tubes) reduce hasta en un 14% los resultados de PTH en comparación con los tubos habituales de separación de suero (SST, del inglés serum separator tubes), posiblemente ocasionado por cambios producidos por la trombina sobre la PTH que modifican su antigenicidad. Finalmente, también influye la zona donde se realiza la extracción de la muestra; las muestras obtenidas de sangre proveniente de una vía central registran resultados hasta un 30% más elevados que los cuantificados en sangre periférica [6]. Esto es importante ya que, aunque lo habitual es realizar la venopunción y extracción de sangre periférica, hay situaciones especiales donde la muestra procede de sangre central, como en pacientes en hemodiálisis o en la cirugía de paratiroides, y sería conveniente especificar la zona de extracción para interpretar correctamente los resultados, especialmente cuando se realizan mediciones seriadas de PTH.

## Variabilidad analítica de la PTH

Añadiéndose a estos factores preanalíticos, en el caso particular de la PTH, destaca su enorme variabilidad analítica con importantes diferencias en los resultados dependiendo del método utilizado para su cuantificación [8]; una situación que pasa desapercibida a priori porque los intervalos de referencia aportados por los diferentes proveedores son prácticamente superponibles. Diversos factores explican esta importante variabilidad inter-método que se detecta en la cuantificación de la PTH. Entre ellos, merecen mención especial, en primer lugar, la gran heterogeneidad de sus péptidos circulantes y en segundo, las diferencias, tanto en la configuración antigénica (lo que implica que no todos los métodos reconozcan las mismas formas circulantes), como en el origen de los calibradores con que los diferentes fabricantes estandarizaron cada uno de los inmunoensayos comerciales disponibles.

### Heterogeneidad de los péptidos circulantes de la PTH

**Figura 1:**
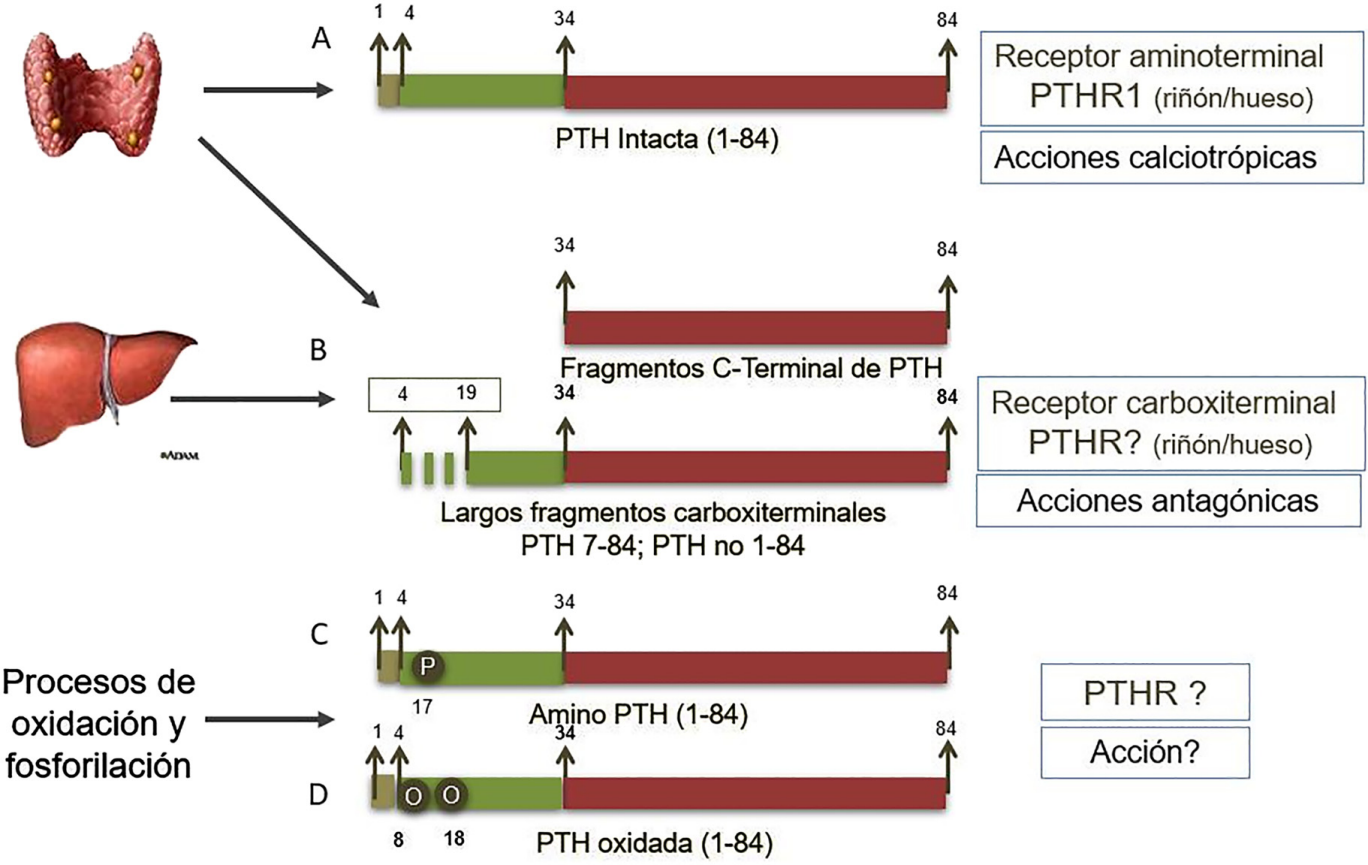
Péptidos circulantes de la PTH. La PTH circulante es una mezcla de: (A) PTH intacta 1–84, biológicamente activa, liberada en respuesta a la hipocalcemia para incrementar los niveles circulantes de calcio tras activar al receptor 1 (PTHR1); (B) Péptidos carboxiterminales resultantes de la degradación intraglandular y hepática de la PTH 1–84, de los cuales alrededor de un 10% corresponden a largos péptidos escindidos entre los aminoácidos 4 y 19 (PTH truncada a nivel aminoterminal, PTH 7–84 ó PTH no 1–84) y con efecto antagónico al de la PTH 1–84 al activar un receptor PTH carboxiterminal aun no identificado; (C) Amino PTH, PTH 1–34 fosforilada con función desconocida y (D) PTH oxidada en los aminoácidos 8 y 18, procesos postraslacionales que interfieren en su unión al PTHR1.

La PTH circulante (Figura 1) es una mezcla de (a) *PTH intacta 1–84*, biológicamente activa, liberada por la glándula paratiroidea en respuesta a la hipocalcemia para incrementar los niveles circulantes de calcio mediante la activación aminoterminal del receptor 1 de la PTH (PTHR1) en riñón y hueso, y (b) una serie de *péptidos carboxiterminales*, fruto de la degradación intraglandular y hepática de la PTH 1–84. Alrededor de un 10% de estos fragmentos de PTH corresponden a largos péptidos escindidos entre los aminoácidos 4 y 19, y que conocemos como PTH truncada a nivel aminoterminal, PTH 7–84 ó PTH no 1–84 [9]; curiosamente, estos fragmentos no son inactivos, sino que ejercen un efecto antagónico al de la PTH 1–84 mediante la activación de un receptor PTH carboxiterminal aún no identificado [10]. Complicando todavía más estas formas circulantes, la PTH sufre *procesos post-translacionales de oxidación* en los residuos metionina situados en los aminoácidos 8 y 18 [11] y *de fosforilación,* especialmente en el residuo serina del aminoácido 17, conocida como “amino-PTH” y que se sobrexpresa en diversas patologías, como el carcinoma de paratiroides [12]; ambas modificaciones limitan la capacidad de la PTH 1–84 para activar el PTHR1.

### Diferencias entre los métodos de medida de PTH

**Figura 2:**
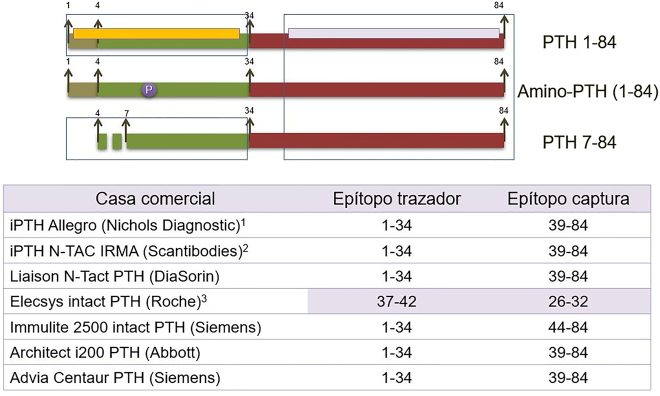
Métodos de segunda generación. Mal denominados “métodos de PTH intacta” ya que, en su día, se interpretó que cuantificaban exclusivamente la PTH 1–84 biológicamente activa (aún no se había identificado la PTH 7–84). Estos largos fragmentos, con acciones antagónicas a la PTH 1–84, se aclaran por el riñón; situación que hay que tener en cuenta cuando se utilizan en la ERC. (1) Método ya desaparecido validado por histomorfometría ósea con el que se hicieron la mayoría de las guías nefrológicas. (2) Método IRMA isotópico. (3) Método de PTH intacta que muestra una configuración diferente al resto de CLIAs automatizados. Presenta también interferencia con la amino-PTH ya que su anticuerpo aminoterminal (curiosamente el de captura) es muy distal.

**Figura 3:**
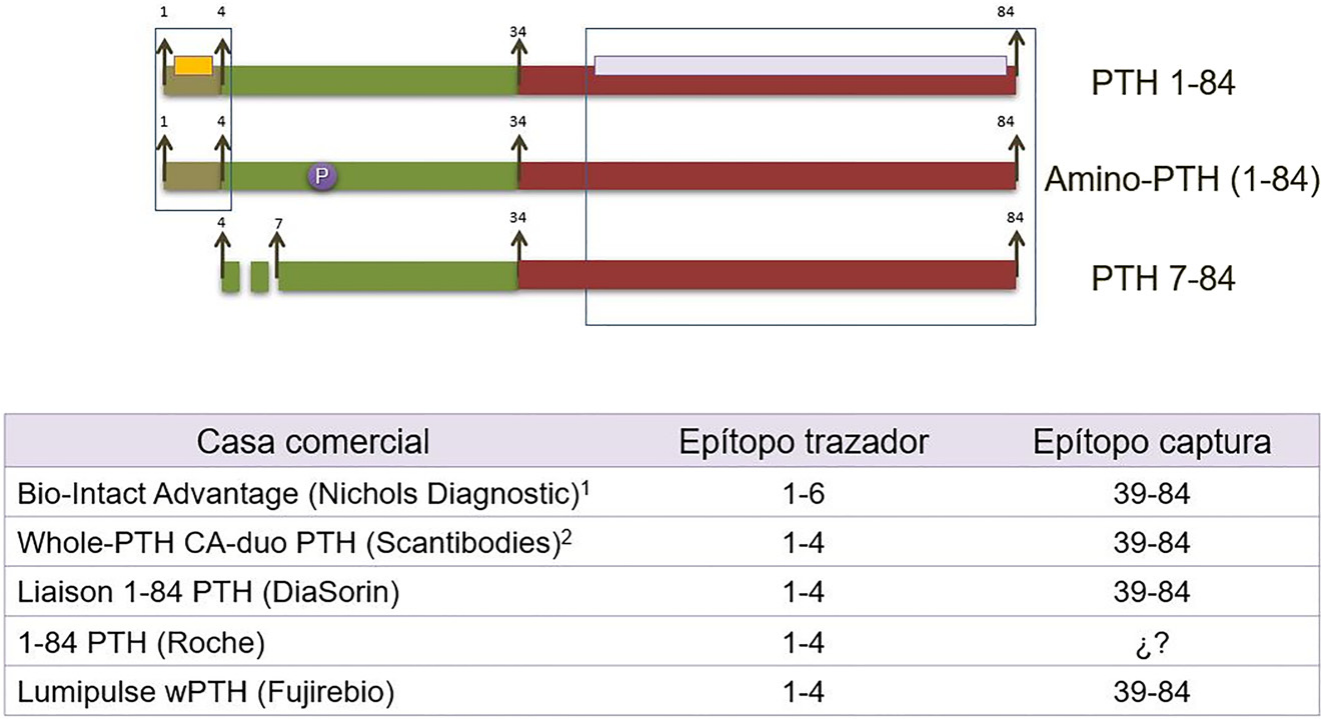
Métodos de tercera generación. Denominados como métodos de PTH bio-intacta, se desarrollaron para evitar la interferencia de la PTH 7–84 y con ellos, la cuantificación por ello de efectos antagónicos de la PTH. Para ello, su epítopo trazador reconoce exclusivamente los 4–6 primeros aminoácidos del extremo aminoterminal. (1) Dejo de fabricarse como consecuencia de importantes problemas de precisión, que acabaron con la desaparición del proveedor. (2) Método IRMA isotópico no automatizado.

La segunda causa de variabilidad inter-método responde a las diferencias en la configuración antigénica entre los diferentes métodos de medida de PTH. La *primera generación* de métodos, que se desarrollaron hacia 1980, eran radioinmunoanálisis (RIA) competitivos, poco sensibles y poco específicos, que utilizaban un solo anticuerpo dirigido generalmente contra las regiones carboxiterminal o media, de modo que cuantificaban tanto PTH 1–84 como todas sus formas circulantes. En 1987 aparecieron los métodos de *segunda generación* (Figura 2) [13], [14] que utilizan dos anticuerpos, generalmente, uno carboxiterminal que inmoviliza la molécula y un segundo dirigido contra la región amino terminal que está marcado y permite su cuantificación. Se conocen como “métodos de PTH intacta” (i-PTH) porque, hasta la identificación de la PTH 7–84 [9], se creía que se cuantificaba exclusivamente la PTH 1–84 biológicamente activa. Actualmente se sigue utilizando esta denominación inadecuada a pesar de que, como consecuencia de esta interferencia con la PTH 7–84, estamos cuantificando efectos antagónicos de la hormona. Por ello, para evitar esta situación surgieron los métodos de *tercera generación*, conocidos como PTH biointacta (bio-PTH) o PTH-whole (Figura 3), que se diferencian de los anteriores en que el segundo anticuerpo reconoce únicamente los 4–5 primeros aminoácidos de la molécula [15]; de este modo, no cuantifica la PTH 7–84 aunque sí las formas modificadas postraslacionalmente de la PTH.

Teniendo en cuenta estas diferencias en la configuración antigénica, son lógicas las discrepancias de resultados entre los métodos de 2^a^ y 3^a^ generación, ya que cuantifican diferentes péptidos circulantes. Sin embargo, resulta sorprendente la existencia de importantes diferencias entre los propios métodos de una misma generación [16], llegándose a observar desviaciones desde −40% hasta 120%, entre los diversos inmunoensayos comerciales de i-PTH cuando se referencian frente al IRMA i-PTH utilizado para establecer las primeras guías de práctica clínica de la *International Kidney Foundation* [17]. Esta alarmante situación se debe a que, en su día, los diferentes fabricantes calibraron sus métodos de i-PTH, frente a distintos estándares con orígenes muy diversos (bovino, murino…) con la consiguiente falta de conmutabilidad [8], [16], al no existir entonces el actual estándar internacional de PTH 1–84 humana recombinante.

## Repercusión de la variabilidad analítica de la PTH en la enfermedad renal

La identificación de estos problemas que plantean los métodos de medida de i-PTH alertó sobre posibles errores en la interpretación de sus resultados, y especialmente en el paciente con enfermedad renal, donde la medición de la PTH constituye una herramienta diagnóstica básica para evaluar la afectación del metabolismo mineral en la ERC. La pérdida de nefronas y de la expresión renal de klotho, el incremento adaptativo del factor de crecimiento fibroblástico 23 (FGF23) y la inhibición de la vitamina D con su correspondiente hipocalcemia, desencadenan el desarrollo de un marcado hiperparatiroidismo secundario que ocasiona importantes anomalías esqueléticas. Estas alteraciones óseas se recogen bajo el término específico de osteodistrofia renal y engloban situaciones de bajo y alto remodelado. La PTH es el test de elección en el diagnóstico no invasivo de esta osteodistrofia, ya que el *gold estándar,* que es el estudio histomorfométrico tras doble marcaje con tetraciclinas en biopsia ósea de cresta iliaca, es inviable en la práctica clínica. Es más, a pesar de la reciente identificación de nuevos marcadores mas precoces y sensibles para evaluar el deterioro en la homeóstasis mineral en la ERC, como el FGF23 y el klotho, la mayoría de las guías [17], [18] siguen basando sus decisiones terapéuticas en los niveles circulantes de la PTH. El problema que plantea la utilización de inmunoensayos i-PTH, específicamente en la población renal, es que los fragmentos carboxiterminales se aclaran por el riñón, de modo que la proporción de PTH 7–84 aumenta conforme disminuye el filtrado glomerular (FG), alcanzando aproximadamente un 50% de las formas circulantes de PTH en el estadio 5 de ERC. Este inconveniente no afecta a otras patologías como el hiperparatiroidismo primario (originado por hiperplasia, adenoma o carcinoma, de una o varias glándulas paratiroideas) u otras causas de hiperparatiroidismo secundario (ej tras cirugía bariátrica o en situaciones de hipovitaminosis D), donde el incremento en los niveles de PTH circulante no es tan acusado y el porcentaje de PTH 7–84, al no acumularse por existir una función renal normal, es menos relevante.

**Figura 4:**
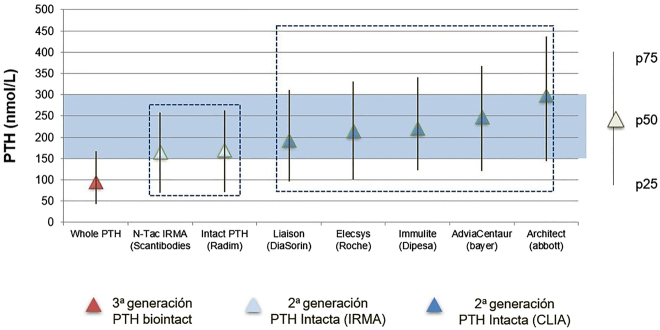
Diferencias en la distribución por percentiles de una población de pacientes con enfermedad renal crónica estadio 5 prevalente en hemodiálisis entre los diferentes métodos de PTH (estudio SEN*). Los resultados obtenidos con el método de PTH biointacta son lógicamente inferiores a los registrados con los métodos de PTH intacta, ya que no cuantifican la PTH 7–84. Sin embargo, también se observan importantes diferencias entre los métodos de 2^a^ generación, obteniéndose valores mas bajos con los métodos isotópicos IRMA que con los CLIA automatizados; es mas, dentro de estos últimos sorprenden esas discrepancias y en particular, las medianas y percentiles registrados en la plataforma Architect (Abbott). *Datos procedentes del estudio de la SEN [18].

El reconocimiento de esta variabilidad en los resultados, dependiendo del método i-PTH utilizado en el manejo clínico del paciente renal, replanteó la validez de guías clínicas como las “*National Kidney Foundation/Kidney Dialysis Outcomes Quality Initiative*” (NKF/KDOQI) [17], que establecía unos intervalos de i-PTH para cada estadio de ERC en base a la correlación de un método de segunda generación (iPTH IRMA Allegro*; Nichols Institute Diagnostics Inc*) con la histomorfometría en biopsias óseas. De hecho, las sucesivas guías “*Kidney Disease – Improving Global Outcomes*” (KDIGO) [18], editadas con posterioridad, recogen esta situación y recomiendan rangos mucho mas amplios (entre 2 y 9 veces el límite superior del rango de normalidad (Upper Normal Limit, [UNL] establecido para cada método). Por su parte, la Sociedad Española de Nefrología (SEN) diseñó una serie de estudios para establecer posibles equivalencias entre los inmunoensayos i-PTH más utilizados en nuestro país y en relación al único método bio-PTH disponible en ese momento [19]. Se registraron resultados mas elevados con los métodos i-PTH automatizados quimioluminescentes (CLIA) que con los inmunorradiométricos (IRMAs) e incluso importantes diferencias entre los propios CLIA que afectaban a los criterios de decisión clínica (Figuras 4 y 5). Afortunadamente, la excelente correlación entre todos los inmunoensayos evaluados permitió establecer unos algoritmos de ajuste inter-método y, para facilitar la aplicación de las guías KDOQI en los pacientes con ERC estadio 5, se diseñaron unas tarjetas con los datos de equivalencia [19]. Es importante puntualizar que estos algoritmos iniciales de la SEN se establecieron en una población de ERC estadio 5 prevalente en hemodiálisis (HD), donde el acúmulo de la PTH 7–84 es máximo. Ello implica que, estos algoritmos de equivalencia inter-método no eran aplicables en estadios previos de ERC, donde la proporción de fragmentos carboxiterminales es menor [20]. Es mas, también se demostraron diferencias en el porcentaje de formas circulantes de PTH dependiendo del tipo de diálisis. Para un mismo valor de i-PTH, la proporción de PTH 1–84 en los pacientes en diálisis peritoneal (DP) era inferior a la registrada en HD, como consecuencia del mayor porcentaje de calcio ionizado circulante por la pérdida de proteínas a través del peritoneo [21]. Esta situación justifica la mayor prevalencia de enfermedad adinámica del hueso observada en DP frente a HD y, es sumamente importante ya que, al aplicar en DP los algoritmos del estudio inicial en HD en vez de los específicos para DP [22], podríamos “adinamizar” iatrogénicamente aún más a estos pacientes. En cualquier caso, la solución del problema de esta variabilidad inter-método de la PTH, que afecta especialmente a la ERC, no debería ser la utilización de algoritmos de ajuste sino la exigencia de una adecuada estandarización.

**Figura 5:**
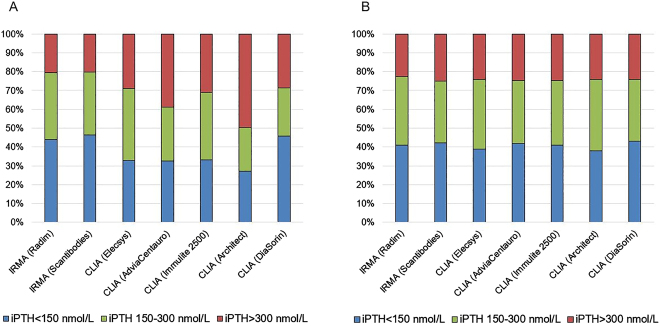
Diferencias en la categorización de los pacientes con ERC 5 prevalentes en hemodiálisis por criterios KDOQI en función del método de iPTH utilizado (estudio SEN*). Las guías KDOQI recomiendan un intervalo de PTH entre 150 y 300 nmol/L para los pacientes con estadio 5 de ERC en diálisis, basado en estudios correlación del desaparecido método isotópico de iPTH “Allegro” (Nichols Institute) con histomorfometría ósea. Los niveles circulantes de iPTH menores de 150 nmol/L sugieren bajo remodelado óseo, mientras que los valores superiores a 300 nmol/L indicarían alto remodelado. (A) Al estratificar a los pacientes según los resultados de iPTH obtenidos “en crudo” se observan importantes diferencias en los porcentajes de cada categoría, especialmente con el CLIA Architect (Abbott) según el cual, la mitad de los pacientes se etiquetarían como alto remodelo y serían subsidiarios de tratamiento con agentes antiparatiroideos. (B) Cuando se aplican los correspondientes algoritmos de ajuste del estudio SEN para hemodiálisis [18], se observa que solamente un 25% aproximado de los pacientes precisaría la corrección del hiperparatiroidismo y se habrían tomado decisiones clínicas erróneas. *Datos procedentes del estudio de la SEN [18].

## Posición de los laboratorios clínicos

Ante este escenario, la utilización en el abordaje clínico del paciente renal de los métodos de tercera generación para medición de PTH resulta esperanzadora. Sin embargo, hasta hace unos años, el único método disponible de tercera generación era el método IRMA dual (CA-PTH Duo Scantibodies) que incluía un ensayo de i-PTH y otro de bio-PTH [23]; el ser un método isotópico, unido a la falta de automatización y su elevado precio, impidió su introducción en el laboratorio clínico. Tiempo atrás, Nichols Institute Diagnostics Inc había diseñado un método quimioluminescente (CLIA) automatizado de bio-PTH (Nichols Advantage Bio-Intact PTH) que se implementó en muchos laboratorios, pero importantes problemas de precisión [24] entre los años 2003 y 2005, con desviaciones de hasta +29 y +52%, terminaron con la quiebra económica de este proveedor (y con ella, la desaparición de la i-PTH Allegro con que se referenciaron las KDOQI) un año después. Esto supuso el regreso a los métodos i-PTH, que son los que actualmente se utilizan en la gran mayoría de los laboratorios clínicos, aun sabiendo que cuantifican efectos antagónicos de la hormona y reconociendo sus limitaciones. De hecho, las KDIGO siguen recomendando la utilización de los métodos de segunda generación por su disponibilidad y difusión en todos los laboratorios clínicos.

### Los métodos de PTH biointacta en la osteodistrofia renal

Afortunadamente, en la actualidad ya disponemos de nuevos métodos automatizados de bio-PTH que nos ofrecen la oportunidad de recuperar ese “paso atrás” [25]. Su implementación a nivel clínico, nos permite aproximarnos nuevamente a la realidad de la molécula biológicamente activa. Es más, ocasionalmente, la cuantificación conjunta de i-PTH y bio-PTH también podría aportar información adicional en la evaluación de la osteodistrofia renal. Este sería el caso del diagnóstico de bajo remodelado óseo, situación en la que existen mayores discrepancias entre los valores de PTH y la histomorfometría ósea [26]. Con el ánimo de minimizar estos posibles errores diagnósticos del remodelado óseo, algunos autores proponen otras alternativas para expresar los resultados de PTH, como el cociente PTH 1–84/PTH 7–84 [27] o incluso la combinación de este ratio con la i-PTH (cuyos valores<420 pg/mL asociados a una ratio PTH 1–84/PTH 7–84<1, en pacientes ERC 5D de raza blanca, serian predictivos de bajo remodelado óseo con una sensibilidad del 90%) [28]. El inconveniente de la utilización de estas ratios es que se asume que la concentración de PTH 7–84 equivale a la diferencia entre la i-PTH y la bio-PTH respectivamente. Sin embargo, esta consideración no es correcta al existir formas oxidadas o fosforiladas circulantes de PTH, como la amino-PTH, que presenta reactividad cruzada con los métodos bio-PTH, pero no se detecta con la mayoría de los inmunoensayos i-PTH cuyos anticuerpos identifican el segmento amimoterminal que incluye el aminoácido fosforilado. Como contrapartida, esto podría tener utilidad teórica en el diagnóstico y monitorización de aquellas patologías en las que se sobreexpresa la amino-PTH [12]. Al ser menor la proporción de amino-PTH que la del fragmento 7–84, en condiciones fisiológicas, el cociente bio-PTH /i-PTH [(*PTH 1–84* + *aminoPTH*)*/*(*PTH 1–84* + *PTH 7–84*)] debería ser inferior a 1 (exceptuando el método i-PTH de Roche cuyos epítopos son: 26–32 y 37–42 y cuantifica PTH 1–84 + PTH-7–84 + amino-PTH); la inversión de este cociente podría identificar un posible carcinoma de paratiroides entre aquellos pacientes con hiperparatiroidismo primario [29], [30]. Análogamente, en el contexto del hiperparatiroidismo secundario en la ERC, la sobreexpresión de la amino-PTH y la inversión del cociente podría reflejar una marcada hiperplasia paratiroidea, indicando el tratamiento con Cinacalcet (fármaco calcimimético que, al incrementar la sensibilidad del receptor sensor de calcio de la glándula paratiroidea para el calcio, se utiliza en la corrección del hiperparatiroidismo secundario) o la realización de una paratiroidectomía [31].

Independientemente de la posible utilidad de estas ratios, la realidad es que los métodos de bio-PTH permitirían una mejor estandarización y comparación de los pacientes con ERC frente a esa población sana de referencia, sin la interferencia de la PTH 7–84 cuya concentración varia de forma notable dependiendo de la tasa de filtrado glomerular, tipo de diálisis, etc.,. Con ello estaríamos reduciendo incertidumbre biológica a la medición, a pesar de la reactividad cruzada con la PTH oxidada y con la amino-PTH. No hay que olvidar que la PTH oxidada se incrementa en el paciente renal por stress oxidativo y que las metioninas oxidadas afectan a la interacción de la PTH con el receptor PTHR1, perdiendo actividad; de hecho, el reciente desarrollo de un método para medir PTH oxidada nos permitirá conocer su significado fisiopatológico y su implicación clínica [11], [32].

### Estandarización de los métodos de PTH biointacta

Adicionalmente, la estandarización clínica de los métodos de tercera generación ofrece la oportunidad de corregir y evitar definitivamente esos innecesarios y peligrosos ajustes inter-método, exigiendo la calibración todos los inmunoensayos comerciales de PTH frente a un *gold estándar* universal como referencia. En octubre de 2009, el Comité de Expertos sobre estandarizaciones biológicas de la OMS estableció el primer estándar internacional humano recombinante para PTH 1–84 (WHO International Standard; NIBSC code: 95/646). A raíz de esto, algunos grupos [33] comienzan a referenciar los métodos automatizados de bio-PTH existentes en ese momento (Roche Elecsys y DiaSorin Liaison) y calibrados con otros estándares, frente a ese estándar internacional humano recombinante, de modo que tras la re-estandarización desaparecen las posibles diferencias inter-método y se establecen nuevos intervalos de referencia biológicos (IRB). En línea con este criterio, proveedores como Fujirebio, desarrollan posteriormente su método automatizado de bio-PTH calibrándolo ya frente al estándar NIBSC code 95/646. La IFCC, sensible a esta problemática, crea un grupo de expertos en PTH con el objetivo de mejorar la comparabilidad en los resultados de PTH [6]. Entre sus líneas de trabajo está precisamente exigir la calibración de los diferentes métodos frente a este estándar internacional y definir el método de referencia para su cuantificación, siendo el tándem cromatografía liquida/espectrometría de masas (LCMS/MS), el actual candidato pendiente de optimización [34].

En relación a las especificaciones de calidad a seleccionar, en la 1^a^ Conferencia Estratégica de la *European Federation of Clinical Chemistry and Laboratory Medicine* (EFLM), celebrada en Milán en 2014, se consensuó la validez del modelo jerárquico de Estocolmo, simplificándolo en 3 modelos: especificaciones basadas en el impacto clínico, variación biológica y el estado del arte. A falta de estudios que revelen el impacto clínico de la variabilidad analítica en la toma de decisiones clínicas, la variación biológica constituye la estrategia más enfocada al uso clínico de la PTH. Por otra parte, la EFLM a través del grupo de trabajo sobre Variación Biológica (VB), junto al *Task Group* para el desarrollo de la base de datos de VB, desarrolla una herramienta, “*critical appraisal checklist*” (BIVAC) [35] y simultáneamente, siguiendo ese estándar, diseña y lleva a cabo un estudio experimental multicéntrico europeo denominado EUBIVAs para estudiar la VB de múltiples magnitudes, entre los que se encuentra la bio-PTH [36] con resultados de estimados de coeficiente de variación intraindividual (CV_I_) de 14.7 (IC 95%: 14.0–15.5), mientras que el estimado de CV_I_ resultante del metanálisis realizado por este grupo derivado del resto de estudios existentes para la PTH (https://biologicalvariation.eu/bv_specifications/measurand#) es de 24.2 (IC 95%: 20.2–25.9).

## Posible adaptación futura de los laboratorios clínicos sobre la medición de la PTH para aproximarse a la realidad del paciente con ERC

A pesar de todos estos argumentos, los inmunoensayos de tercera generación no terminan de introducirse en la práctica diaria para el manejo del paciente renal. El principal problema que dificulta su implementación a nivel clínico es la experiencia limitada y la ausencia de guías con este tipo de ensayos (para el clínico, lo que no esta en guías clínicas no existe, aunque ofrezca beneficios). Su estandarización frente a la osteodistrofia resulta compleja si tenemos en cuenta que la biopsia e histomorfometría ósea es un método poco accesible, invasivo y caro. Algunos estudios asocian la bio-PTH del inmunoensayo isotópico de Scantibodies con biopsias óseas, como el anteriormente mencionado de Herberth sobre ratios [28], pero sobretodo disponemos de datos indirectos a través de su referenciación y ajuste con la i-PTH “Allegro” de las guías KDOQI [8]. Según este estudio, el intervalo de 150–300 pg/mL de i-PTH, recomendado por las KDOQI [17] para estadio ERC 5HD, corresponde a 84–165 pg/mL de bio-PTH Scantibodies [8]. En cualquier caso, *a priori,* la adaptación de la bio-PTH a las recomendaciones KDOQI para los estadios iniciales de ERC no es compleja, ya que siguiendo el criterio de las guías que sugieren valores de i-PTH dentro de intervalos de referencia de la población supuestamente sana, los valores de bio-PTH deberían adecuarse al IRB. Mas problemático resulta definir el rango para el estadio 4, cuando las guías establecen unos valores de 70–110 pg/mL de iPTH. Sin embargo, aun reconociendo la contribución de la PTH oxidada, creemos que esta discreta elevación en la i-PTH obedece fundamentalmente al acúmulo de los largos fragmentos carboxi-terminales por la reducción del filtrado glomerular y, por tanto, se debería intentar mantener la bio-PTH en rangos aproximados a los estadios anteriores por carecer de reactividad cruzada con estos fragmentos. No obstante, no podemos olvidar que la hipovitaminosis D tiene una prevalencia muy elevada en la ERC, mayor aun que en la población general, y que esta situación tiene un importante impacto en los niveles circulantes de PTH 1–84.

Partiendo de esta aproximación, es un objetivo prioritario conseguir una estandarización adecuada de la PTH para la población renal, utilizando los métodos de tercera generación. Aunque las guías KDOQI, pensadas inicialmente para el diagnóstico no invasivo de la afectación ósea en la ERC, mantienen su vigencia, el conocimiento de las alteraciones en el metabolismo mineral que subyacen en la ERC nos sitúa actualmente en otro escenario de trabajo. En el año 2006, el grupo de trabajo de KDIGO [37] amplia el concepto clásico osteodistrofia renal (término que queda específicamente restringido para las alteraciones óseas de la ERC) y lo sustituye por la denominación de CKD/MBD (por sus siglas del inglés: *Chronic Kidney Disease – Mineral and Bone Disorders*) para remarcar la precocidad de estas alteraciones (desde el minuto 1 de la ERC) y fundamentalmente la afectación de otros tejidos extra-óseos, especialmente del sistema cardiovascular, principal causa de mortalidad del paciente renal. De este modo, el objetivo de tratamiento de estas alteraciones va más allá de la corrección del hiperparatiroidismo y de la osteodistrofia renal, y persigue otros beneficios, especialmente sobre la progresión de la enfermedad renal y la mortalidad cardiovascular. Desde este nuevo punto de vista, y más aun con las limitaciones de los estudios óseos, los rangos de referencia para la bio-PTH no deberían establecerse en función de la distribución y comparación de resultados entre la población sana y los diferentes estadios de ERC, sobretodo cuando estamos valorando mecanismos adaptativos. La evaluación clínica de la bio-PTH debe fundamentarse en el diseño de estudios robustos de supervivencia que evalúen tanto mortalidad como progresión a la diálisis. Con este criterio, existe un estudio reciente que evalúa los niveles circulantes de PTH 1–84 (DiaSorin) asociados a estos efectos adversos en 1812 pacientes renales con un filtrado glomerular estimado (eFG) entre 15 y 45 mL/min [38] y con una media de seguimiento de 52 meses. La mayoría de los pacientes muestran valores de PTH en sangre por encima del límite superior (ULN: 39.4 pg/mL), duplicándose este valor en los pacientes con eFG inferior a 20 mL/min. El umbral de riesgo basado en los eventos cardiovasculares que encuentran es 42.9 pg/mL para eFG≥30 mL/min; 104.6 pg/mL para eFG 20–29 mL/min y 134 pg/mL para eFG<20 mL/min; siendo los umbrales de riesgo basado en progresión a la diálisis renal: 53.5 pg/mL para eFG ≥ 30 mL/min; 49.4 pg/mL para eFG 20–29 mL/min y 93.7 pg/mL para eFG<20 mL/min.

Queda aun mucho trabajo por delante y muchos aspectos aún por conocer de la PTH y sus formas circulantes. Aun así, creemos que es el momento de comenzar a utilizar los métodos de tercera generación, de evaluar su utilidad clínica y demostrar que esta ventaja teórica sobre los métodos i-PTH facilita realmente la monitorización de la función de la paratiroides y especialmente la toma de decisiones clínicas en la ERC. Los nuevos avances en el conocimiento de los mecanismos etiopatogénicos que subyacen en las alteraciones de la homeostasis mineral en la ERC, deben encontrar también su respuesta en el laboratorio clínico, de modo que la cuantificación de la bio-PTH y del FGF23 [39] en sangre debieran constituir una herramienta diagnóstica básica en el abordaje de la CKD/MBD. Como profesionales del Laboratorio clínico debemos impulsar iniciativas de trabajo en colaboración con otros especialistas clínicos cuyo objetivo sea mejorar la calidad de nuestros resultados. Ahora o nunca.
